# Important parameters for optimized metal nanoparticles-aided electromagnetic field (EMF) effect on cancer

**DOI:** 10.1186/s12645-018-0038-4

**Published:** 2018-03-15

**Authors:** Lawrence Ochoo, Charles Migwi, John Okumu

**Affiliations:** Physics Department, Kenyatta University, Box 43844, Nairobi, 00100 Kenya

**Keywords:** Cancer, Metal nanoparticles, Electromagnetic field, Photothermal therapy, Electric field effect

## Abstract

**Background:**

A number of experimental research findings for the metal nanoparticles (NPs)-mediated EMF photothermal therapy of cancer cells show an intriguing trend of the NPs’ size-dependent efficacy. This is a phenomenon we find to trend with the light absorption bandwidth behavior (full width at half maximum) of the NPs and the accompanying electric field enhancement. We find that the nanoparticle sizes that have been reported to produce the optimized effect on cancer cells are of minimum absorption bandwidth and optimized electric field magnitude. While the death of cancer cells under the NPs-aided EMF effect has in the past attracted varied interpretations, either as a thermal or non-thermal effect, photothermal effect has gained a wide acceptance due to the exhibited hyperthermia. However, the exhibited trend of the NPs’ size-dependent efficacy is beginning to feature as a possible manifestation of other overlooked underlying or synergistic phenomenal conditions.

**Method:**

We present a theoretical model and analysis which reveal that the contribution and efficacy of the metal NPs in the destruction of cancer depend partly but significantly on the accompanying electric field intensity enhancement factor and partly on their absorption cross-section.

**Results:**

This paper finds that, other than the expected hyperthermia, the metal NPs’ sizes for the optimized therapy on cancer cells seem to fulfill other synergistic conditions which need to come to the fore. We find interplay between electric field and thermal effects as independent energy channels where balancing may be important for the optimized EMF effect, in the ratio of about 5:1. The required balancing depends on the absorption bandwidth and absorption cross-section of the NPs, the frequency of EMF used and the relative permittivity of the cancer cells. The NPs’ size-dependent efficacy decreases away from the NPs’ size of minimum absorption bandwidth, which is around 20 nm for Au NPs or other shapes of equivalent surface area–volume ratio. While the absorption wavelength peak for metal NPs would change with the change of shape, the responsible condition(s) for optimizing the efficacy remains relatively invariable.

**Conclusion:**

From the modeling and the analysis of the NPs’ size for optimizing the EMF therapy on cancer cells, the ratio of electric field enhancement by metal NPs to the associated thermal effect is a very important factor for efficacy.

## Background

Cancer can be viewed as an abnormal phenomenon in a human cell, where the afflicted cells are unable to balance their uptake of the necessary nutrients, uncontrollably grow and subdivide endlessly without dying like the normal cells. The desire to control this endless cell division and spread has been tried through single treatment mechanism(s) and sometimes a combination of two or more therapies such as surgical, chemotherapy, radiotherapy and biological mechanisms. The quest for mechanisms that would selectively correct or destroy cancer cells has led to researches that attempt to explore novel ideas involving nanoparticles-based thermal therapies. For example, electromagnetic field (EMF)-induced thermotherapy with Au metal or magnetic nanoparticles, in combination with chemotherapy or radiotherapy and chemotherapy and the like, has been proposed (Chen et al. 2015). It is beginning to emerge that the treatment of cancer cells may benefit more from a combination of various forms of therapy. However, the underlying mechanisms and their contributions to efficiency and risks are still debatable. For example, chemotherapy and radiotherapy ionizing radiations are linked to irreversible damage on the DNA of a cell; therefore, their use may have beneficial effect in the cancer cell therapy (Mi et al. 2016). The common fear, however, has been how such damages can be controlled not to extend to the normal cells. New technological dispensations, promising the use of nanoparticles (size 1–100 nm) with multiple effects (photothermal, electrical and magnetic) which can be switched on selectively, are being investigated (Liu et al. 2014; Mi et al. 2016). Here, we focus on the issue of the condition(s) imposed by the Au NPs’ sizes for the optimized photothermal therapy of cancer cells. There is substantial evidence that, in the past two decades, the interpretation of the experimental results has varied between the thermal (temperature) and non-thermal effects, almost in equal measure (El-Sayed et al. 2006; Li and Gu 2010). Of particular interest is that the metal NPs-mediated EMF therapy of cancer cells revealed an unprecedented NPs’ size-dependent trend. The most efficient Au NPs’ sizes have been reported to be within 10–30 nm range, with the efficacy reducing outside the range toward the smaller or the larger NP sizes (Zharov et al. 2005; Mackey et al. 2014). In this paper, we observe that within this size range, the variation of the optical absorption bandwidth for the Au NPs full width at half maximum (FWHM) with the NPs’ size (below 50 nm) produces a curve with a minimum turning point. The turning point is found to coincide with the median value for the size range (10–30 nm) and it corresponds to the Au NPs’ size reported to show the optimized effect on cancer cells. From the experimental results of Link and El-Sayed (1999a), the minimum absorption bandwidth for Au is visibly at the NPs’ size of 21.7 nm. Link and El-Sayed (1999a, b, 2000) had initially reported 40-nm Au NPs to be effective in the destruction of cancer cells, before Zharov et al. (2003, 2005) reported the limiting effective NPs’ size range (10–30 nm). However, there is an intriguing issue that this range of NPs’ sizes exhibits the same trend of influence on the dye/solar cell efficiency (Photiphitak et al. 2010). Based on our previously proposed model for the absorption of light by metal NPs below 50 nm (Ochoo et al. 2012), both the Au and Ag NPs produce their turning points within the 10–30 nm range. In both cases of the cancer cells and dye/solar cells, the efficacy seems to trend with the NPs’ size-dependent absorption bandwidth behavior. This is suggestive of an underlying common NPs’ size and absorption bandwidth-dependent phenomenon, whose role in optimizing both thermal and non-thermal effects requires the same condition.

The dilemma is that the NPs-mediated EMF therapy seems to induce numerous inseparable effects, each of which has been associated with the death of cancer cells (Zharov et al. 2003, 2005; Day et al. 2009). Experimental reports have demonstrated that Au NPs can convert laser energy (UV-NIR) into heat and cause hyperthermia, in picoseconds (Iancu 2013; Yao et al. 2016). The reports of thermal effect seem to have overshadowed the thought that a possibility of any other underlying phenomena being essential in the NPs-EMF destruction of cancer cells be its complementary or synergistic contribution. According to Zharov et al. (2003, 2005), on irradiating the Au NPs with pulsed laser the cell damage is induced through a series of non-thermal phenomena such as shock waves, cavitations and structure rupturing. They were of the view that the temperature merely initiates the generation of microbubbles around the NPs, which are the primary cause of the cavitation damage of the cancer cells. This argument may appear to gain credence in relation to other reports. For example, according to Li and Gu (2010), temperature only serves to amplify non-thermal mechanical damage of the cancer cells. Generally, cancer cells have higher water content than the normal cells; therefore, the reports of observed microbubbles around Au NPs, which have been associated with the cavitation damage of the cancer cells, could be suggestive of a frothing fluid (Zharov et al. 2005; Iancu 2013). Whether the microbubbles and their “claimed effect” are a consequence of thermal or other non-thermal effects is not established. They could as well be a product of electrolytic process, since human cells interact with a variety of ions of imbalanced concentrations on opposite sides of the membrane (K^+^, Na^+^, Ca^2+^) (Lobikin et al. 2012; Persinger and Lafrenie 2014). The thermal effect, according to Choi et al. (2011), is supposed to be dependent on its severity, influenced by thermal intensity and duration at high temperatures. Leung et al. (2013) have also reported that cancer exhibits high thermotolerance to hyperthermia and, if unassisted, would require higher temperatures for effective treatment. Experimental studies have reported that the laser power required to kill cancer cells can be as low as ~ 0.5 W/cm^2^ when aided with Au NPs, especially Au nanorods, compared to about 1600 W/cm^2^ when unaided (Iancu 2013). It is intriguing that the metal NPs could be making cancer cells less thermotolerant, behaving like a catalytic effect. Also, why they should be NPs of size around 20 nm, and where the absorption bandwidth tends to its minimum (turning point), would be of interest.

In a recent report, it was observed that for a clinical application of Au NPs as a radiosensitizer, it would be beneficial to know the role of NPs’ sizes or shapes (Yao et al. 2016). Majority of reports, however, only emphasize the potential or successes of various NPs’ shapes or design (nanoshells, nanorods, nanorings, etc.) in the destruction of cancer cells. The assumption is that the responsible mechanism and condition are already known (hyperthermia). Unfortunately, little has been reported about the range of metal NPs’ sizes of various shapes, and the conditions under which they optimize the destruction of cancer cells by laser other than the threshold temperatures. It has been explained that the NPs’ size is only a factor in balancing between the cell uptake and retention of the NPs, as a condition for efficient thermal therapy (Choi et al. 2011). This has contributed to an ad hoc system of choosing NPs’ sizes for trial tests on cancer cells. In the transformation of light energy by the NPs, heat is considered to be generating very rapidly within the metal NPs (lattice), by the electron–electron and electron–phonon interactions. The lattice then loses the energy in about 100 ps, through the phonon–phonon relaxation, which heats up the surrounding medium (Huang and El-Sayed 2010; Choi et al. 2011; Yao et al. 2016). This process suggests that the photothermal process is an electron–phonon–phonon centered phenomenon that would be localized in the particle and, therefore, could depend on the NPs’ size. Interestingly, according to Huang and El-Sayed (2010), the electron–phonon relaxation is size and shape independent. To interrogate the intervening mechanism(s) and the conditions responsible for the optimized effect, not only just the experimental observations but also a sound theoretical model would be necessary for analysis.

In this paper, we introduce a theoretical model that focuses on a relationship between the metal NPs’ sizes and the electric field enhancement around them. It is an alternative model which interrogates the role of the electric field as a phenomenon that acts as an additional energy channel for the metal NPs-aided EMF therapy of cancer. It finds the variation of the electric field intensity enhancement with the NPs’ size to be similar to that of the reported NPs’ size-dependent EMF effect on cancer cells. They both vary inversely with the NPs’ spectral absorption bandwidth. According to the model, the variation of the absorption bandwidth with the NPs’ size exhibits a minimum turning point around the NPs’ sizes that have optimized effect on both the electric field enhancement and the EMF effect on the cancer cells. This suggests that the electric field enhancement by metal NPs could be sharing the same condition(s) with the photothermal effect required for the optimization. Generally, the process of EMF energy transformation by metal NPs is perceived to be through a phenomenon called surface plasmon resonance (SPR). This is where all the free electrons of the NPs are set into oscillation with a uniform frequency by the light’s electric field (Yao et al. 2016). The NPs absorb the EMF energy to sustain the SPR whose part of the energy transfers to the lattice as heat and also enhances electric field around the NPs (Toma et al. 2010; Mackey et al. 2014). While the virtue of the SPR-mediated thermal effect on cancer cells has been extolled and well explained, the enhanced electric field and its bearing on the efficiency of the NPs-mediated EMF effect on cancer cells are just beginning to emerge (Wang et al. 2014). Mackey et al. (2014) have attributed the Au nanorods’ size for the optimized photothermal therapy of cancer cells to energy balancing between heat, electric field and the distance at which the field extends from the NPs’ surfaces. This was based on the observed efficacies of the Au nanorods of lengths 17, 28 and 38 nm, in which the 28-nm sized showed superior effect (Mackey et al. 2014). It was reported that, out of the three NPs sizes, the 17- and 28-nm Au nanorods caused equal rise in the temperature (15 °C), but the 28-nm sized still had the superior effect on malignant cancer cells. The report explains that, to optimize the photothermal effect, both the electric field strength and its distance contribute in promoting effective coupling between the NPs. The interesting scenario is that, for a stronger but short-ranged electric fields and weaker but long-ranged fields, as attributed to the 17- and 38-nm nanorods, the NPs do not produce good results even with adequate rise in the temperature. Their experimental and theoretical NPs’ sizes, attributed to appropriate energy balancing, were 28 and 25 nm, respectively. In the light of the emerging potential role of the enhanced electric field on cancer cells, it would be necessary to identify the potential factors influencing this role. Generally, the electric field strength and the temperature effect of NPs would be expected to be influenced by, among other things, the size and shape, absorption cross-section of the NPs and the electric or thermal properties of the host medium. The electric field enhancement factor for metal NPs has been commonly expressed by Eq. 1 (Moores and Goettmann 2006; Toma et al. 2010).

1 $$\frac{{\varvec{E}_{{\text{int}}} }}{{\varvec{E}_{\text{o}} }} = \left( {\frac{{3\varepsilon_{\text{m}} }}{{\varepsilon_{\text{p}} + 2\varepsilon_{\text{m}} }}} \right),$$

where ***E***_int_ is the enhanced electric field around a metal NP, ***E***_o_ is electric field of the incident light, *ε*_p_ and *ε*_m_ are the permittivity of the NPs and the medium, respectively.

Equation 1, however, suggests that the electric field enhancement would be majorly dependent on the permittivity of the host medium ($\varepsilon_{\text{m}}$), especially when the permittivity of the NPs becomes relatively negligible (*ε*_p_ ≪ *ε*_m_). As a result, it would be inadequate for explaining the contributions or the roles of factors such as the NPs’ size, shape, metal type and their absorption characteristics to the thermal or electric field (***E***_int_) effects on the cancer cells. For example, some studies have tried to analyze the effect of externally applied AC electric fields on cancer cells, with the aim of determining optimal frequencies that can hinder cell proliferation (Schwab et al. 2012; Hondroulis et al. 2014). The potential effect of the external electric field on cancer cells has also been linked to the electrical properties of the cells, through the bioelectric interaction of the cytoplasm with the extracellular environment. It has been reported that by manipulating the electric potential gradient between the cytoplasm and the extracellular environment (*V*_m_), making it more negative (hyperpolarizing), the division of cancer cells is blocked in the *V*_m_ range of − 45 to − 75 mV (Schwab et al. 2012; Persinger and Lafrenie 2014). The cancer cell cycle resumes on depolarizing the membrane to around − 10 mV. The manipulation of *V*_m_ by an alternating electric field has been associated with the field effect on the distribution of the essential ions in the cytoplasm and the cell membrane (Lobikin et al. 2012; Persinger and Lafrenie 2014). Because *V*_m_ is critical for the permeability of different ions and the cell functions, its modulation would certainly influence the cell behavior. Given that *V*_m_ is responsive to the artificial alternating electric field, the effect of alternating electric field around the metal NPs to the efficicacy of EMF on the cancer cells may be interpreted in the same light. This can introduce an interesting dimension in the management of the recurring cancers, that a periodic hyperpolarization of *V*_m_ would be an alternative remedy for the patients. The report of Mackey et al. (2014) detailing that the most appropriate nanorod size (28 nm) was due to proper balancing between the electric field strength, field range and heating rate would require further analysis. It could be a consequence of the response of *V*_m_ to the alternating field. The sensitivity of the cancer cell membrane to electric field has been reported to be dependent on the frequency of the field (Hondroulis et al. 2014), in a manner we find similar to the NPs’ size effect. In this paper, we show that the condition(s) characterizing the NPs’ size-dependent efficacy of EMF therapy of cancer cells can be derived from the parameters for the SPR-triggered electric field. The SPR presents itself as an absorption peak whose location and bandwidth are dependent on the metal type, NPs’ sizes and shape, the medium and the wavelength of light used (Ochoo et al. 2012; Pinchuk et al. 2004). As a result, the SPR-triggered electric field and its associated effect or role in the EMF therapy would be expected to be influenced by the same factors.

### Metal nanoparticles’ optical properties and efficiency

The photothermal effect of the metal NPs and their electric field strength enhancement would be expected to manifest in the absorption bandwidth. A broadening bandwidth would be expected to lead to enhanced heating effect while a narrowing bandwidth should enhance the induced electric field strength. The broadband absorption suggests a wide range of frequencies of the electronic oscillations, causing incoherence and increased electron collisions and, hence, the heating effect. Thus, the bandwidth broadening would be expected to favor a high yield in the EMF energy conversion to heat. Ag NPs usually exhibit the narrowest absorption bands in the UV range of 390–430 nm while Au and Cu NPs produce broader bands in the visible range of 520–590 nm (Huang and El-Sayed 2010). Elsewhere, gold nanoshells have been reported to exhibit broader bandwidth and higher heat content, leading to higher photothermal conversion compared to the nanorods but their effects on cancer cells are either the same or the nanorods perform better (Choi et al. 2011; Yao et al. 2016). A comparison of Au NPs of different shapes in the destruction of cancer cells, in the NIR region, has revealed that nanorods are about six times more efficient than the nanoshells and nanospheres (Choi et al. 2011; Popp et al. 2014; Fang et al. 2015; Robatjazi et al. 2015). Why the nanoshells, whose absorption bandwidth is broader, do not translate into the most efficient photothermal agent for cancer therapy is not yet fully understood. The difference has since been attributed not to the conversion yield but to the rapidity of energy conversion rate by the nanorods (Choi et al. 2011; Yao et al. 2016). Factors that make the SPR of the nanorod have a higher conversion rate than the other shapes, and even why certain nanorods do better than others, are not fully explored.

## Methods and experiments

### Proposed models for the absorption cross-section of NPs

The Mie (1908) theory, expressed in Eq. 2, has been valuable in explaining the SPR absorption behavior of metal NPs over the years. However, it was later found to be inconsistent with the NPs’ size-dependent absorption bandwidth variation, which exhibits a minimum turning point (Kawabata and Kubo 1966; Kreibig and Genzel 1985). This inconsistency was attributed to possible factors such as a chemical effect (acts as catalyst between interacting molecules), quantum effect and the use of dielectric constants for the bulk metal instead of the NPs’ size-dependent values (Kawabata and Kubo 1966; Kreibig and Genzel 1985).

2 $$\sigma_{{\text{abs}}} = \frac{{24\pi^{2} R^{3} \varepsilon_{\text{m}}^{3/2} }}{{\lambda_{{\rm max} } }}\frac{{\varepsilon_{2} }}{{(\varepsilon_{1} + 2\varepsilon_{\text{m}} )^{2} + \varepsilon_{2}^{2} }},$$

where *σ*_abs_ is the absorption cross-section of NPs, *R* is the radius of a spherical nanoparticle, *ε*_m_ is the dielectric constant of the medium, *λ*_max_ is the wavelength of absorption peak, *ε*_1_ and *ε*_2_ are the real and imaginary parts of the dielectric constants of the bulk metal. For small *ε*_2_, absorption peak is realized when *ε*_1_ = − 2*ε*_m_.

The inconsistency of Eq. 2, in its failure to produce the absorption bandwidth behavior with a turning point, makes it inadequate for probing the parameters of influence around the turning point. Therefore, we introduce our earlier model, (Eq. 3) (Ochoo et al. 2012), whose form and parameters are consistent with the NPs’ absorption bandwidth behavior. We use it to interrogate the occurrence of a turning point (minimum absorption bandwidth), as a factor of influence for the optimization of the efficiency of NPs-aided EMF effect on cancer cells. It is much similar to Eq. 2, but with additional parameters.

3 $$\sigma_{{\text{abs}}} = \frac{{18\pi R^{3} \varepsilon_{\text{m}}^{3/2} }}{{\lambda_{0} }}\left[ {\frac{{Ze\rho \varepsilon_{\text{p}}^{1/4} }}{m}} \right]^{2} \left[ {\frac{{\varepsilon_{\text{p}} - \varepsilon_{\text{m}} }}{{\varepsilon_{\text{p}} + 2\varepsilon_{\text{m}} }}} \right]^{2} \frac{1}{{\left[ {\eta_{x}^{2} + \eta_{y}^{2} + \eta_{z}^{2} } \right]^{1/2} }}\frac{1}{{\left[ \omega \right]^{{\prime }} }},$$

where the symbols have same meanings as those in Eq. 2, *Z* is atomic number of a metal, *e*/*m* is electronic charge–mass ratio, *ρ* is charge density of a metal, *ε*_p_ is imaginary part of the dielectric constant for NPs, *n* is the order of oscillatory mode, [*ω*]^*′*^ = (*ω*_p_^2^ − *ω*^2^)^2^ + *γ*^2^*ω*^2^, *ω*_p_ = 2*πc*/*λ*_p_, *c* is speed of light, *ω*_p_ and *λ*_p_ are NPs’ plasmon resonance frequency and wavelength, and *ω* is the frequency of incident light.

**Fig. 1:**
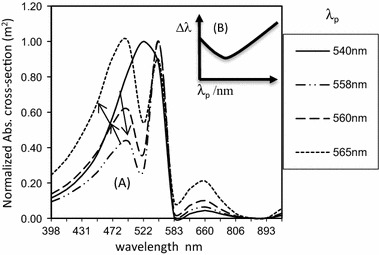
Variation of absorption bandwidth at various excited SPR frequencies corresponding to Plasmon wavelengths *λ*_p_

Similar to the Mie theory, the dielectric constants for bulk metals are used in Eq. 3. Here, the imaginary part of the permittivity for the metals, from the Johnson and Christy (1972), is used for *ε*_p_. Figure 1 shows the model’s normalized spectral absorption cross-section and the bandwidth (FWHM) behavior for Au NPs.

Figure 1 shows that as the excitation wavelength (*λ*_p_) for the SPR increases, the single broadband absorption peak splits into three peaks, causing the narrowing of absorption bandwidth to a minimum (turning point) then broadening. This is depicted in the inscribed curve. From this prediction, Eq. 3 suggests that the inadequacy of the Mie theory about the turning point may not have been a consequence of the use of dielectric constants for the bulk metals. It is noted that there is excitation wavelength (*λ*_p_ = *λ*_min_ = 568 nm) for which the minimum absorption bandwidth occurs, just as in the NPs’ size effect. This wavelength is expected to correspond to the Au NPs’ size for the minimum absorption bandwidth. Also, as in the case of NPs’ size-dependent peak position, the SPR absorption peaks in Fig. 1 shift within the wavelength range 520–585 nm, corresponding to the occurrence of the SPR peaks for Au NPs’ sizes below 50 nm (Qian and Park 2010; Link and El-Sayed 1999a). The additional peaks showing around 480 and 660 nm do not shift, and they correspond to the interband and intraband absorption for Au, respectively (Qian and Park 2010). These positive predictions, especially the bandwidth behavior, make the parameters of Eq. 3 potential for interrogating the role of the NPs’ sizes whose absorption peaks occur around *λ*_min_ (turning point). In this paper, our point of reference is how the NPs’ sizes with minimum absorption bandwidth (*λ*_min_) relate to the optimizing effect of the EMF on the cancer cells.

### Correlation between metal NPs’ shapes with optimized efficiency

The use of different shapes of NPs such as nanoshells and nanorods, for cancer therapy, has basically been an attempt to shift their absorption peaks to the wavelengths where the body tissues could be transparent to allow in vivo application(Huang and El-Sayed 2010; Cherukuri et al. 2010). This could suggest that the metal NPs showing optimized effect on cancer cells and dye/solar cells, in all shapes, may be related and the shape transformation may only be to improve other aspects. This could be true only if the optimizing properties of the NPs were dependent on the mechanism(s) and parameters that do not change on shape transformation. That is, the nanosphere sizes for optimized cancer treatment can be transformed into nanoshells or nanorods with expected similar results. Here, we propose the surface area (SA) and surface area–volume ratio (SA/VR) as the parameters of equivalence for relating NPs with similar effects on cancer but of different shapes. This is based on the premise that SPR absorption is a surface phenomenon. Table 1 shows a comparison of the SA and SA/VR, for few nanospheres, nanorods and nanoshells of dimensions reported in other studies to be of efficient EMF effect on cancer cells and dye/solar cells (Zharov et al. 2005; Photiphitak et al. 2010; Choi et al. 2011; Iancu 2013).

**Table 1:** Calculated estimates of surface area and surface area–volume ratios of common NP shapes and sizes used in cancer therapy, equivalent of nanospheres of 10–30 nm size

Shape	Size (diameter) (nm)	SA (m^2^)	Volume (m^3^)	SA/V-R
Nanospheres	10	3.14 × 10^−16^	5.23 × 10^−25^	6.00 × 10^8^
	20	12.56 × 10^−16^	41.87 × 10^−25^	3.00 × 10^8^
	21.7^a^	14.79 × 10^−16^	53.48 × 10^−25^	2.77 × 10^8^
	30	28.26 × 10^−16^	141.3 × 10^−25^	2.00 × 10^8^
	40	50.24 × 10^−16^	334.93 × 10^−25^	1.50 × 10^8^
Nanorods^b^	*d* = 5, *l* = 17	3.06 × 10^−16^	3.34 × 10^−25^	9.16 × 10^8^
	*d* = 8, *l* = 28	8.03 × 10^−16^	14.07 × 10^−25^	5.71 × 10^8^
	*d* = 11, *l* = 38	13.38 × 10^−16^	36.9 × 10^−25^	3.71 × 10^8^
Nanoshells	*C* = 30, *S* = 8	66.4 × 10^−16^	53.1 × 10^−24^	1.25 × 10^8^
	*C* = 120, S = 5	530.7 × 10^−16^	265.30 × 10^−24^	2.00 × 10^8^
	C = 100, *S* = 7.5	415.3 × 10^−16^	311.5 × 10^−24^	1.33 × 10^8^

*d* diameter, *l* length, *C* core diameter, *S* Au shell thickness

^a^Au NP size at minimum absorption bandwidth (Link and El-Sayed 1999a)

^b^Experimental nanorod sizes used for photothermal therapy (Mackey et al. 2014)

The SA and SA/VR values in Table 1 have been estimated by simple mathematical formulae for regular-shaped objects, not considering atomic sizes and packing. The estimated SA/VR results for the nanorods and nanospheres (10–30 nm) with optimizing effect on cancer and dye/solar cells are seen to correspond well. This suggests that SA/VR can be a parameter for the transformation of the efficient nanospheres to efficient nanorods. Photiphitak et al. (2010), using Ag NPs in the size range (3.66–38.5 nm) on dye/solar cell(s), reported the most effective size to be 19.2 nm. Also, for Ag/Si-based photovoltaic cell the optimized results have been obtained with 20-nm Ag NPs (Hu and Chen 2008). Similarly, Au of size 22 nm and Ag NPs of 31.4 nm have been reported to optimize the absorption of light by methylene blue dye (Uppal et al. 2011). Thus, the NPs’ size effect on the dye/solar cells is a replica of what is to be anticipated with cancer cells. Almost all the NPs’ sizes fall around that of the minimum absorption bandwidth (turning point), which is around 21.7 nm for the Au NPs (Link and El-Sayed 1999a). For Ag NPs, the occurrence of a turning point has not been clearly established from the previous studies. From the experimental results of Bijanzadeh et al. (2012), there is no turning point exhibited for Ag NPs in the size range of 2–34 nm. Suggesting that either the NPs do not exhibit a turning point or it could be outside this size range. In the report of Slistan-Grijatra et al. (2005), it is only stated as being within the 20–40 nm range. Because of the bearing of turning point on the NPs’ sizes for optimizing the EMF effect on cancer, we have determined it experimentally for Ag and theoretically (Ag and Au). We conducted an experimental measurement on Ag NPs in the size range 9–44 nm, on glass substrates. The NPs were prepared by irradiating AgNO_3_ in ethanol with a 366-nm UV light source.

## Results and discussion

### Experimental analysis of minimum absorption bandwidth for Ag

**Fig. 2:**
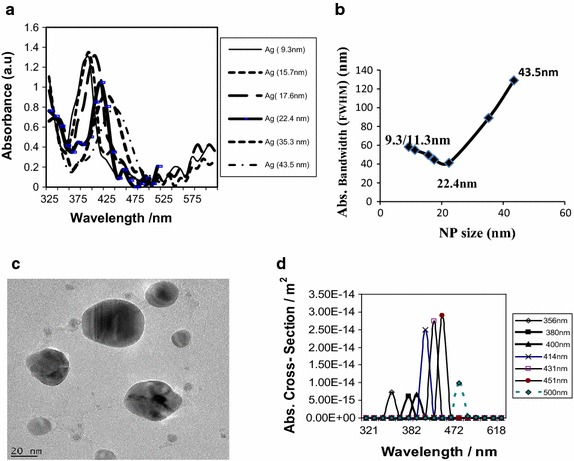
Ag NPs: (**a**) Experimental spectra (**b**) variation of the absorption bandwidth (FWHM) with NP size. **c** TEM sample for the 22.4-nm Ag NPs at minimum absorption bandwidth in **b**. **d** Theoretical curves computed from Eq. 3

Figure 2a, b shows the experimental results for the variation of absorption bandwidth for Ag NPs over the NPs’ size range of 9–44 nm, and the minimum absorption bandwidth (FWHM) occurs at the average NPs’ size of 22.4 nm.

The experimental and theoretical spectra for Ag (Fig. 2a, d) correlate well; they do not exhibit as much overlap of the absorption modes as in Au NPs (Fig. 1). This could be attributed to low damping effect on the SPR of Ag NPs, compared to the Au and Cu NPs (Ochoo et al. 2012). Of most significance here is that the turning point for Ag, like Au NPs, also falls within the anticipated NPs’ size range 10–30 nm, where the Au NPs’ sizes with the optimizing effect on both the cancer cells and the dye/solar cells fall.

### Calculated NPs’ sizes of minimum absorption bandwidth

Equation (4), derivable from Eq. (3) (Ochoo et al. 2012), is used here to confirm and validate the actual metal Au, Ag and Cu NPs’ sizes of the minimum absorption bandwidths.

4 $$\Delta \lambda = a\frac{{\gamma \lambda_{{\text{max}}}^{2} }}{2\pi c} + b\frac{{\lambda_{{\text{max}}}^{2} \pi kh}}{{4\pi R^{2} mc}},$$

where Δ*λ* is the absorption bandwidth of NPs, *λ*_max_ is wavelength of the peak location, *γ* is (bulk) damping constant term, *c* is speed of light, *h* is Planck’s constant, *R* is the radius of NPs, *m* is mass of electron, $$m = \left( {n_{x}^{2} + n_{y}^{2} + n_{z}^{2} } \right)^{{{\raise0.7ex\hbox{$1$} \!\mathord{\left/ {\vphantom {1 2}}\right.\kern-0pt} \!\lower0.7ex\hbox{$2$}}}}$$, *c* is speed of light, ‘*a*’ and ‘*b*’ are constants (39.2 and − 25.4, respectively) obtained through the experimental data for gold from Bac et al. (2011).

The first term on the right-hand side is dependent on the damping term (*γ*) for the bulk material. The second term, however, is inversely dependent on the NPs’ size by the surface area equivalent of a nanosphere (4*πR*^2^). Thus, the two terms should represent the right and left sides of the turning point of the curve, respectively (Figs. 1, 2b). In respect of this, at the turning point the condition for continuity is expected to be fulfilled by the two terms of Eq. 4. That is, the laws governing the plasmon resonance behavior on the right and left sides coincide at the turning point and should be equal. This would be true when Δ*λ* is set to zero. Thus, unless there are external damping factors on the NPs’ absorption bandwidth, the absorption should approximate to a spectral line (Δ*λ* = 0). With this condition, the spherical NPs’ sizes with minima absorption bandwidths would be given by Eq. 5.

5 $$R_{\text{o}} = \sqrt {\left( {\frac{{\left( { - b} \right)}}{{\left( { a } \right)}}\frac{\pi kh}{2m\gamma }} \right)} ,$$

where *R*_o_ is the radius of a spherical particle of the minimum absorption bandwidth.

From Eq. 5, *R*_o_ is independent of the wavelength of the absorbed light but is inversely dependent on the damping term (*γ*). To compute *R*_o_, the experimental values for (*γ*), from Johnson and Christy (1972), have been used here. For gold *γ* is 0.072 eV (1.69 × 10^13^/s); for Cu, *γ* is (2.31 × 10^13^/s) and for Ag, *γ* is 0.021 eV (5.04 × 10^12^/s). Because Au and Cu exhibit higher orders of SPR modes/damping (multipolar), we take *k* = 3 while for Ag *k* = 1 due to low modes (dipolar). The diameter size (2*R*_o_) of the spherical Au NPs is found to be 21.7 nm, the same as from the experimental result of Link and El-Sayed (2000). For Cu, the calculated size is 19.7 nm and for Ag it is 24.2 nm. These values are in good agreement with the experimental values, therefore, confirming that the NPs’ sizes for the minimum absorption bandwidths lie within the 10–30 nm range. The corresponding approximate sizes in other shapes may be worked out based on the SA/VR equivalence. SA or SA/VR as the parameter for good performance would elicit the thought of SA-dependent phenomena such as the catalytic effect (chemical), heating effect (temperature) and electric field effect (charge density). The concern in this paper is about the phenomena that would justify the influence of the minimum absorption bandwidths on the metal NPs’ sizes of different shapes in the EMF therapy. In the photothermal effect theory, the limiting NPs’ size range for optimized results has been attributed to NPs uptake, distribution and retention by the cells (Choi et al. 2011). This perspective may lack scientific justification for the case of dyes and solar cells, whose enhanced light absorption optimizes at the same NPs’ sizes of Ag and Au as for the cancer cells (Photiphitak et al. 2010; Uppal et al. 2011). On the other hand, a catalytic effect would be expected to increase for the reducing NPs’ size (< 10 nm) because of the increasing SA/VR. However, unlike for the most effective NPs’ size (around 20 nm), they exhibit broader absorption bandwidths. It is the electric field (***E***) effect that appears to identify positively with the characteristics and parameters in question, especially the issue of the minimum absorption bandwidth.

### Electric field enhancement by metal nanoparticles

For the purpose of this paper, the electric field enhancement (***E***_int_**/*E***_o_) by metal NPs would be considered in the light of the parameters of Eq. 6, and the source of Eqs. (3, 4, 5) derived in a previous report (Ochoo et al. 2012). In comparison to Eq. 1, the expression of Eq. 6 includes the frequency of light, the permittivity of the medium and NPs, and nuclear charge (Ze), among other parameters. By Eq. 6, the electric field enhancement would be tunable and would optimize at resonance, when the denominator fulfills either condition *ω* = *ω*_p_ or *ε*_p_ = − 2*ε*_m_.

6 $$\frac{{\varvec{E}_{{\text{int}}} }}{{\varvec{E}_{\text{o}} }} = \left( {\frac{{3\varepsilon_{\text{m}} }}{{\varepsilon_{\text{p}} + 2\varepsilon_{\text{m}} }}} \right)\left( {\frac{{\text{Ze}\rho }}{m}} \right)\left( {\frac{{\varepsilon_{\text{p}} - \varepsilon_{\text{m}} }}{{\left[ {\left( {\omega_{\text{p}}^{2} - \omega^{2} } \right)^{2} + \gamma^{2} \omega^{2} } \right]^{{{\raise0.7ex\hbox{$1$} \!\mathord{\left/ {\vphantom {1 2}}\right.\kern-0pt} \!\lower0.7ex\hbox{$2$}}}} }}} \right)$$

The resonance condition is easy to achieve by varying *ω* to match *ω*_p_, Eq. 6 then reduces to Eq. 7. Thus, the electric field enhancement and its potential effect would depend on the metal type, the difference between the permittivity of the NPs and the medium (*ε*_p_ − *ε*_m_) plus factors that may influence *ω*_p_ such as the NPs’ size, shape and the medium.

7 $$\frac{{\varvec{E}_{{\text{int}}} }}{{\varvec{E}_{\text{o}} }} = \left( {\frac{{3\varepsilon_{\text{m}} }}{{\varepsilon_{\text{p}} + 2\varepsilon_{\text{m}} }}} \right)\left( {\frac{{\text{Ze}\rho }}{m}} \right)\left( {\varepsilon_{\text{p}} - \varepsilon_{\text{m}} } \right)\left( {\frac{1}{{ \gamma \omega_{\text{p}} }}} \right)$$

Because of the negative sign in the term (ε_p_ − ε_m_), the magnitude of the permittivity parameter (*ε*_p_) may or may not impact on the magnitude of ***E***_int_. For the medium where *ε*_p_ ≪ *ε*_m_, Eq. 7 approximates to Eq. 8 and ***E***_int_ becomes independent of *ε*_p_. Because cancer cells have higher water content than the normal cells, their measured dielectric constant (*ε*_r_) values are about 64 (in vivo) and 62 (ex vivo) (Cho et al. 2006). Therefore, the approximation of Eq. 8 would fit the purpose of this paper. It predicts the amplification of the EMF’s electric field by a factor that would depend inversely on the SPR frequency (*ω*_p_). The negative sign indicates that ***E***_int_ would be opposite to the incident field ***E***_o_.

8 $$\varvec{E}_{{\text{int}}} = - \frac{{3\varepsilon_{\text{m}} }}{2}\frac{{\left( {\text{Ze}\rho } \right)}}{m}\frac{1}{{\gamma \omega_{\text{p}} }}\varvec{E}_{\text{o}}$$

From the Johnson and Christy (1972), the imaginary values for *ε*_p_ vary with the light wavelength, from about 2.0 in the absorption wavelength range of 520–985 nm for Au NPs to about 25.0 at the wavelength of 1939 nm. Because of the highly reduced frequency (*ω*_p_) at the wavelength of 1939 nm, ***E***_int_ would increase but not much (about 1.2 times) relative to region 520–985 nm. This suggests that the variation of *ε*_p_ does not influence the enhancement factor significantly. In a further analysis, we incorporate the influence of the spectral absorption bandwidth (Δ*λ*) by introducing it into Eqs. 7 or 8, through the parameter (*γ*). Here, we restrict ourselves to Eq. 8 for a while. Equation 9 gives the expression for (*γ*), from Eq. 4. Because the damping term (*γ*) is a property of the bulk material, it suffices to ignore the size-dependent term in Eq. 9 (second term). This leads to Eq. 10, with Δ*λ* and *λ*_max_ as the optical parameters which can be obtained from the absorption spectra of the metal NPs.

9 $$\gamma = \frac{2\pi c\Delta \lambda }{{a\lambda_{{\text{max}}}^{2} }} - \left( {\frac{b}{a}} \right)\frac{{2\pi^{2} kh}}{{4\pi R^{2} m}}$$

10 $$\varvec{E}_{{\text{int}}} = - \frac{{3\varepsilon_{\text{m}} }}{2}\frac{{\left( {\text{Ze}\rho } \right)}}{m}\frac{1}{{\omega_{p} }}\frac{{a \lambda_{{\text{max}}}^{2} }}{2\pi c\Delta \lambda }\varvec{E}_{\text{o}}$$

A parameter for nanosphere size (*R*) can be re-introduced through the de Broglie wave equation (*λ* = *h*/*μ*) and associated quantum expression for the kinetic energy of a free particle in a 3D box model, where the 1D energy model takes the form *E*_k_ = *n*^2^*h*^2^/8 m(2*R*)^2^.

At resonance, let $\omega = \omega_{\text{p}} = \frac{2\pi c}{{\lambda_{\text{p}} }}$ and set *λ*_p_ = *h*/*μ*; therefore,

11 $$\omega_{\text{p}} = 2\pi c\frac{\mu }{h} .$$

From the classical expression for kinetic energy of a free particle, $E_{\text{k}} = \frac{1}{2}\mu^{2}$, hence, $\omega_{\text{p}} = 2\pi c\frac{{\sqrt {2mE_{\text{k}} } }}{h}$, where $E_{\text{k}} = \frac{{h^{2} }}{{8m\left( {2R} \right)^{2} }}\left( {n_{x}^{2} + n_{y}^{2} + n_{z}^{2} } \right)$ for a 3D box model. Hence,

12 $$\omega_{\text{p}} = \frac{k\pi c }{2R},\;\text{where}\;k = \left( {n_{x}^{2} + n_{y}^{2} + n_{z}^{2} } \right)^{{{\raise0.7ex\hbox{$1$} \!\mathord{\left/ {\vphantom {1 2}}\right.\kern-0pt} \!\lower0.7ex\hbox{$2$}}}} .$$

Thus, Eqs. 10 and 12 can lead to Eq. 13.

13 $$\frac{{\varvec{E}_{{\text{int}}} }}{{\varvec{E}_{\text{O}} }} = - \frac{{3\varepsilon_{\text{m}} }}{2}\left( {\frac{{\text{Ze}\rho }}{m}} \right)\left( {\frac{a}{{\left( {2\pi c} \right) ^{2} }}} \right)\frac{{ (4R) \lambda_{{\text{max}}}^{2} }}{k \Delta \lambda }$$

Or, by setting $2c = \omega \lambda_{{\text{max}}}$ at resonance (Eq. 11), Eq. 13 can be expressed as in Eq. 14.

14 $$\frac{{\varvec{E}_{{\text{int}}} }}{{\varvec{E}_{\text{O}} }} = - 3\varepsilon_{\text{m}} \left( {\frac{{\text{Ze}\rho }}{m}} \right) \frac{a}{{\omega^{2} }}\frac{{ \left( {2R} \right) }}{k \Delta \lambda }$$

**Fig. 3:**
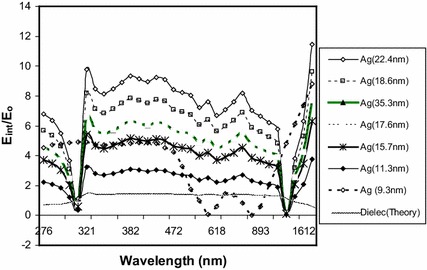
Calculated enhancement of electric field *E*_int_/*E*_o_ for AgNP, Eqs. 6 and 1 dielectric (theory)

By Eq. 14, for a given excitation light of frequency (*ω*), the electric field enhancement would be influenced by the ratio of the NPs’ size to the absorption bandwidth (2*R*/Δ*λ*), optimizing at the minimum value of Δ*λ* (Eq. 14). This would correspond to the turning point of the absorption bandwidth curves (Figs. 1 and 2b). As the NPs’ size (2*R*) or the excitation wavelength *λ*_p_ increases from left toward the turning point (Figs. 1 and 2b), Δ*λ* decreases then increases after the turning point. Thus, the enhancement of ***E***_int_ is expected to decrease in either direction away from the turning point. Figure 3 shows the calculated electric field enhancement according to Eqs. 1 and 6, for the experimental Ag nanoparticles’ sizes 9–34 nm whose spectra are presented in Fig. 2a.

Figure 3 reveals that the optimized magnitude of ***E***_int_ occurs at the average NPs’ size of 22.4 nm, which is the size at the minimum absorption bandwidth (turning point) in Fig. 2b. As the Ag NPs’ size shifts from 22.4 nm, the enhancement of ***E***_int_ decreases, same trend as in the case of the influence of Au NPs’ size on the efficiency of EMF therapy (Zharov et al. 2003; Mackey et al. 2014). This suggests that the optimizing effect of the NPs’ size on the cancer cells is the same as for the electric field intensity around the NPs. Thus, the way the electric field enhancement follows the trend of the NPs’ size-dependent EMF effect on cancer cells could be suggestive of it having a major role in the EMF therapy. This suggestion would be considered in the context of power (P), to establish how the rate of energy delivered to NPs balances between the enhancement of electric field ***E***_int_ and the dissipation as heat. From the report of El-Sayed et al. (2006), the laser energy (514 nm) kills malignant cells in the presence of Au NPs but not when there is no metal NPs, even at the source power densities four times higher (Giuliani and Soffritti 2010). This implies that the death of cancer cells is caused by the energy delivered to it through conversion by the metal NPs. Because there could be additional conditions, depending on the contributions of the electric field and the NPs’ sizes/shape but not featuring in the parameters of the model, a possible relation between ***E***_int_ and the rate of energy delivery (power) would be discussed below. This is to be done through an alternative mathematical expression, which would bring together parameters of the electric field enhancement and those of the thermal effect. From it, we again find the electric field enhancement of NPs to be an important phenomenon and energy channel, determined by the NPs’ size, optical parameters and condition(s) for the EMF therapy of cancer cells.

### Rate of energy delivered through metal nanoparticles

After the alternating incident electric field of the EMF displaces the free electrons of charge (*q*) in the NPs, storing energy in them, an opposing restoring force associated with ***E***_int_ would set in and dissipate some of the acquired energy during restoration. Depending on the frequency of the incident field and the intervening forces of the medium, the energy balance between the dissipated and that which remains in the system in terms of ***E***_int_ would be dependent on the interacting forces. There are two important forces that would be relevant to this discussion. One is the damping force, it is dependent on the velocity of motion in a medium (*F*_d_), and the other would be the restoring force due to the displacement of the electrons’ cloud from the core ion (*F*_r_). For simplicity, *F*_r_ would be taken to be dependent on the displacement (*x*) and represented as *kx*. These two forces act in opposition to the driving force of the incident ***E***_o_ and are bound to draw and convert the EMF energy into dissipative (thermal) and non-dissipative (electric field), respectively. This kind of interaction can be given by the general equation for damped motion (Eq. 15).

15 $$m\frac{{\text{d}^{2} x}}{{\text{d}t^{2} }} + kx + \beta \frac{{\text{d}x}}{{\text{d}t}} = F \,\text{Cos} \,\omega t$$

$F_{\text{d}} = - \beta \frac{{\text{d}x}}{{\text{d}t}}$ (dissipative force) and $F_{r} = - kx$ restoring non-dissipative force.If the displacement of the free electrons follows the driving force and expressed as *x* = *x*_o_Cos *ωt*, then the restoring force would be *F*_r_ = − *kx*_o_Cos *ωt* while the dissipative force *F*_d_ = *βωx*_o_Sin *ωt*. Therefore, the rate of work done (power) by the restoring force (associated with the NPs-induced electric field) can be expressed by Eq. 16.

16 $$P_{\text{r}} = F_{\text{r}} \frac{{\text{d}x}}{{\text{d}t}} = - q\varvec{E}_{{\text{int}}} \frac{{\text{d}x}}{{\text{d}t}}$$

By introducing Eq. 14 into Eq. 16, we obtain Eq. 17, whose parameters would give the required clue about the factors to influence the system of power delivery by NPs. While the average power value over many cycles (〈*P*_r_〉) would yield zero (Eq. 17), its root mean square value (〈*P*_r_〉_rms_) optimizes at the NPs’ size of minimum absorption bandwidth (Δ*λ*) (Eq. 18).

17 $$P_{\text{r}} = 3\varepsilon_{\text{m}} \left( {\frac{Ze\rho }{m}} \right) \frac{{\left( {2R} \right)a }}{ \omega \Delta \lambda k }q\varvec{E}_{\text{O}} \left( {x_{\text{o}}\, {\text{Sin }}\omega t} \right)$$

18 $$\left\langle {P_{\text{r}} } \right\rangle_{{\text{rms}}} = \frac{3}{2}\varepsilon_{\text{m}} \left( {\frac{Ze\rho }{m}} \right) \frac{{\left( {2R} \right)a }}{ \omega \Delta \lambda k }q\varvec{E}_{\text{O}} x_{\text{o}}$$

Since the displacement amplitude (*x*_o_) of the electrons is proportional to the displacing field ***E***_o_, Eq. 18 can be expressed as Eq. 19.

19 $$\left\langle {P_{\text{r}} } \right\rangle_{{\text{rms}}} = \frac{3}{2}\varepsilon_{\text{m}} \left( {\frac{{\text{Ze}\rho }}{m}} \right) \frac{{\left( {2R} \right)a }}{ \omega \Delta \lambda k }q \left| {\varvec{E}_{\text{o}} } \right|^{2}$$

Similarly, the average power delivery to the dissipative force (heating) <*P*_d_> would be given by Eq. 20, from Eq. 15. It is dependent directly on the square of the frequency

20 $$\left\langle {P_{\text{d}} } \right\rangle = \frac{1}{2}\beta \left( {\omega x_{\text{o}} } \right)^{2} = \frac{1}{2}\beta \omega^{2} \left| {\varvec{E}_{\text{o}} } \right|^{2}$$

Baffou et al. (2009) proposed the power of heat generation inside NPs by light absorption to be as in Eq. 21. It takes the same form as the corresponding expression for the dissipative force (Eq. 20). It suggests that the rate of EMF energy delivery to the NPs and, possibly, the rate of heating are directly dependent on the absorption cross-section of the NPs (*σ*_abs_), the permittivity of the medium (*ε*_m_) and intensity of the incident electric field.

21 $$\left\langle {P_{\text{d}} } \right\rangle = \frac{{nc\varepsilon_{\text{m}} }}{2}\sigma_{{\text{abs}}} \left| {\varvec{E}_{\text{o}} } \right|^{2} ,$$

where *n* is optical index of the surrounding medium.Assuming the energy delivered to the NPs is shared majorly between the electric field enhancement channel and the thermal conversion channel only, as per Eq. 15, then Eqs. 19 and 21 would represent their respective rates of energy delivery to the NPs and, possibly, to the cancer cells from the EMF. Thus, the overall energy transfer rate (EMF to NPs) would be given as in Eq. 22.

22 $$\left\langle P \right\rangle = \frac{3}{2}\varepsilon_{\text{m}} \left( {\frac{{\text{Ze}\rho }}{m}} \right) \frac{{\left( {2R} \right)a }}{ \omega \Delta \lambda k }q \left| {\varvec{E}_{\text{o}} } \right|^{2} + \frac{{nc\varepsilon_{\text{m}} }}{2}\sigma_{{\text{abs}}} \left| {\varvec{E}_{\text{o}} } \right|^{2}$$

Equation 22 suggests three important issues relevant for the understanding of the role of NPs’ size and their mechanisms of action. The first is that in the absence of the metal NPs such as Au or Ag, the benefit of the electric field channel (first term) would not be realized by the cancer cells. As a result, the EMF effect on cancer cells would be depending solely on the absorption cross-section of the cells (*σ*_abs_), which would be very small. This may explain why, without metal NPs, even the higher energy powers of laser are not effective, suggesting that the thermal channel (heat) for the cells is of insignificant effect. Second, Eq. 22 suggests that the metal NPs do not act through the thermal channel term only, which would be to merely enhance the effective value of *σ*_abs_. It introduces an additional channel (first term) as a likely complementing energy channel. Third, it suggests that it is because of the SPR in metal NPs the rate of energy extraction from EMF to the cancer cells is improved by the additional channel and the enhancement of *σ*_abs_, depending on the magnitudes of the two terms in Eq. 22. If the optimizing effects of the NPs were to be associated with the heating effect only (yield and rate), as attributed to nanoshells and nanorods (Iancu 2013), then the NPs’ size effect would be influenced only by the *σ*_abs_-dependent term (Eq. 22). On the other hand, if the EMF energy is shared between the two channels then the impact of the NPs size would be determined by the role and the magnitude of each of the energy channels in the EMF therapy. That is, both *σ*_abs_ and Δ*λ* would be involved. Since the absorption cross-section of spherical NPs is proportional to the physical surface area [4*πR*^2^ or 2*πR*(2*R*)], then it is proportional to the diameter of the particle. Therefore, the balancing ratio of the energy between the two channels (Eq. 22) would be dependent majorly on the variation of the ratio *σ*_abs_:Δ*λ*, which can be approximated as in Eq. 23.

23 $$\sigma_{\text{abs}} : \Delta \lambda = \frac{{\sigma_{\text{abs }} }}{\Delta \lambda } \cong \frac{2R}{\Delta \lambda }$$

Thus, if the issue of the energy balancing and impact of NPs on the cancer cells is reduced to be the affairs of the diameter of NPs and the absorption bandwidth (2*R*/Δ*λ*), as earlier discussed in Eq. 14, the first term of Eq. 22 (electric field term) becomes very significant in the use of NPs. It is influenced inversely by the variation of Δ*λ*. This could explain why the inclusion of metal NPs in the EMF thermal therapy of cancer cells yields superior results compared to the unaided EMF (*σ*_abs_ only). It would also explain why the NPs-aided EMF effect optimizes as Δ*λ* reduces toward the minimum, which would explain the same trend seen for the NPs’ size-dependent enhancement of the light energy absorption by the dye/solar cells (Photiphitak et al. 2010). Based on the order of the magnitudes of the parameters in the first and the second terms of Eq. 22, for the spherical Au NPs of diameter 22 nm, we get the ratio for the second term to the first term (Eq. 22) to be about 2:5. This suggests that the electric field energy channel takes up an enormous amount of the EMF energy than the thermal channel during irradiation. In the EMF therapy, therefore, a higher electric field energy channel would be expected to induce higher and fast alternating force (impulse or shock) on the membrane and the ions on both sides of the cell membrane. This is likely to influence the redistribution of the ions on either side of the membrane, leading to change in the concentration gradients of these ions and the membrane potential (hyperpolarization). That is, because an electric field is accompanied by electric force whose magnitude is dependent on the charge type, it would act differently on the ions in its vicinity. The common charge carriers found on the cell membrane and in the cytoplasm are Ca^2+^, Na^+^ and K^+^ (Pall 2013). The regions of a cell membrane with the divalent charges, Ca^2+^, Mg^2+^ or Zn^2+^, are likely to experience higher electric force than the monovalent ions (Na^+^ or K^+^). This effect would ultimately alter the functioning of the cancer cells, where the division of cancer cells can be blocked (Lobikin et al. 2012; Persinger and Lafrenie 2014). Also, the penetration depth (range) of the induced alternating electric field (***E***_int_), between the cell membrane and the cytoplasm, would depend on the frequency of the field (***E***_int_). Equation 22 shows the electric field energy-dependent term (first term) to be dependent on the frequency (*ω*) of the EMF inversely. This suggests that while the electric field energy channel of the NPs would be more enhanced at very low frequencies, if other factors are constant, ***E***_int_ and its penetration effect through the membrane would be low due to high capacitive impedance for low frequencies. On the other hand, very high-frequency fields encounter low impedance and would penetrate deeper through the membrane. However, by Eq. 22, very high frequency would lower the energy for the electric field channel (first term). Thus, although the electric field would deliver the higher frequency energy far into the cytoplasm, it would be low energy. This can explain the need for NPs’ sizes of absorption characteristics that can balance the energy intensity and range. Thus, the observed NPs’ size and absorption bandwidth effect on the efficacy of EMF therapy can be interpreted in the light of energy balancing, in which the electric field and thermal channels are complementary. A highly enhanced electric force (*F* = *μq****E***), where *μ* is an enhancement factor, can rupture the cancer cell membrane and cause increased permeability of the plasma membrane, and therefore, allowing into the cell the molecules to which the plasma membrane would be impermeable. This is likely to alter the behavior of the cancer cells and would lead to cell apoptosis. According to Pall (2013), the death of cancer cells can be initiated by the disruption of the plasma membrane, leading to influx of Ca^2+^ which causes cell damage. This is expected to be enhanced by transient effect of electric field as expressed in other studies (Choi et al. 2011). Also, the electric field is likely to cause change in the conformation of the proteins and enzymes embedded within the membrane, which is likely to depend on the strength and range of the field by the NPs’ size used. Elsewhere, laser radiations and pulsed electrical field have been reported to produce similar non-thermal effects on dipolar molecules, like enzymes, without increase in temperatures or dissipation of the energy (Amat et al. 2006). These are findings that agree well with Eq. 22, which suggest the existence of non-thermal-dependent energy channel (electric field). On the basis of the predictions of the proposed model and the existing evidence that the metal NPs-mediated EMF therapy of cancer cells trends with the absorption bandwidth, the enhanced electric field seems to have an important synergistic role to the thermal, as suggested by the model through the terms in Eq. 22.

## Conclusions

By the model and the analysis presented here, we find optimized electric field strength to be a synergistic energy channel in the best performing metal NPs’ sizes toward the EMF therapy on cancer. Based on the parameters of the model, the optimized condition would be achievable with NPs’ sizes of the minimum absorption spectral bandwidth, whose calculated theoretical values we have found to be 19.4, 21.7 and 24.0 nm for the Cu, Au and Ag nanospheres, respectively. These values are evidently within the range described in other reports as the best performing NPs’ size range (10–30 nm), outside which the performance is reported to drop. The calculated electric field enhancement for the Ag NPs (9–34 nm) prepared for this study has been found to optimize at the average size value of about 22.6 nm, whose absorption spectral bandwidth was found to be the minimum in that range of sizes. The finding, that electric field strength is an important requirement for efficient destruction of cancer, is found to be in agreement with the previous report on nanorods; however, the two reports have employed different mathematical models and NPs’ shapes (nanospheres). The point of difference between this report and the other is only on the question of what aspect of electric field is the key. Our model provides different parameters for the direct identification of the NPs’ size for optimized effect on cancer (absorption band width); the other model considers the intensity and distance to which the electric field would extend from the nanoparticles surface. While some of the previous studies have tried to justify the efficacy of metal NPs in the range 10–30 nm in terms of NPs’ size-dependent ability of the cell uptake and retention, we find this interpretation at variance with the established electric field as a phenomenon for enhancing energy extraction from EMF as an improved condition or additional energy channel for best performance. This is irrespective of the NPs’ shape or the region of absorption. Thus, the electric field strength acts as an independent energy channel operating alongside the thermal (heat) channel that provides a two-pronged attack on the cancer cells.
